# Toll-like receptor 2 induced senescence in intervertebral disc cells of patients with back pain can be attenuated by o-vanillin

**DOI:** 10.1186/s13075-021-02504-z

**Published:** 2021-04-16

**Authors:** Matthew Mannarino, Hosni Cherif, Li Li, Kai Sheng, Oded Rabau, Peter Jarzem, Michael H. Weber, Jean A. Ouellet, Lisbet Haglund

**Affiliations:** 1 Department of Surgery, Orthopaedic Research Lab, McGill University, Montreal, Canada; 2 Department of Surgery, McGill Scoliosis and Spine Group, McGill University, Montreal, Canada; 3 Department of Surgery, The Research Institute of McGill University Health Center, Montreal, Canada; 4 Shriner’s Hospital for Children, Montreal, Canada; 5 Department of Surgery, Montreal General Hospital, McGill University Health Centre, Room C9.173,1650 Cedar Ave, Montreal, QC H3G 1A4 Canada

**Keywords:** Intervertebral disc, Senescence, Toll-like receptor 2, o-Vanillin, Degeneration, Inflammation, Senolytics, Back pain

## Abstract

**Background:**

There is an increased level of senescent cells and toll-like teceptor-1, -2, -4, and -6 (TLR) expression in degenerating intervertebral discs (IVDs) from back pain patients. However, it is currently not known if the increase in expression of TLRs is related to the senescent cells or if it is a more general increase on all cells. It is also not known if TLR activation in IVD cells will induce cell senescence.

**Methods:**

Cells from non-degenerate human IVD were obtained from spine donors and cells from degenerate IVDs came from patients undergoing surgery for low back pain. Gene expression of TLR-1,2,4,6, senescence and senescence-associated secretory phenotype (SASP) markers was evaluated by RT-qPCR in isolated cells. Matrix synthesis was verified with safranin-O staining and Dimethyl-Methylene Blue Assay (DMMB) confirmed proteoglycan content. Protein expression of p16^*INK4a*^, SASP factors, and TLR-2 was evaluated by immunocytochemistry (ICC) and/or by enzyme-linked immunosorbent assay (ELISA).

**Results:**

An increase in senescent cells was found following 48-h induction with a TLR-2/6 agonist in cells from both non-degenerate and degenerating human IVDs. Higher levels of SASP factors, TLR-2 gene expression, and protein expression were found following 48-h induction with TLR-2/6 agonist. Treatment with o-vanillin reduced the number of senescent cells, and increased matrix synthesis in IVD cells from back pain patients. Treatment with o-vanillin after induction with TLR-2/6 agonist reduced gene and protein expression of SASP factors and TLR-2. Co-localized staining of p16^*INK4a*^ and TLR-2 demonstrated that senescent cells have a high TLR-2 expression.

**Conclusions:**

Taken together our data demonstrate that activation of TLR-2/6 induce senescence and increase TLR-2 and SASP expression in cells from non-degenerate IVDs of organ donors without degeneration and back pain and in cells from degenerating human IVD of patients with disc degeneration and back pain. The senescent cells showed high TLR-2 expression suggesting a link between TLR activation and cell senescence in human IVD cells. The reduction in senescence, SASP, and TLR-2 expression suggest o-vanillin as a potential disease-modifying drug for patients with disc degeneration and back pain.

## Background

Low back pain is a global health problem that has been associated with intervertebral disc (IVD) degeneration [1–3]. It is experienced by approximately 80% of individuals at some time in their lifespan [4]. Globally, back pain is the number one cause of years lived with disability [4]. The personal costs in reduced quality of life, as well as the economic cost to healthcare systems are enormous and exceeds $100 billion per year in the USA alone [5]. Current evidence suggests that changes in the biomechanical properties of degenerating discs are associated with matrix fragmentation, inflammation, and pain [6]. However, it is less clear how pain and degeneration are initiated and how they could be prevented. There is a growing interest in the accumulation of senescent cells in degenerating and aging tissues. These senescent cells are viable cells that can no longer divide. Senescence can be induced due to the successive shortening of telomere length during replicative cycles [7]. In addition, the number of senescent cells can also be increased by stressors including DNA damaging agents, oxidative stress, mitochondrial dysfunction, load induced injury, and disruption of epigenetic regulation. This phenomenon is called stress-induced premature senescence and it is believed to be linked to the accumulation of senescent cell in degenerate IVDs [8, 9]. Furthermore, senescent cells release an array of inflammatory cytokines, chemokines, and proteases known collectively as the senescence-associated secretory phenotype (SASP) [10].

All senescent cells have common features, but they also possess distinct characteristics which are linked to the different types of senescence (replicative and stress-induced senescence), cell, and tissue types [11, 12]. The inflammatory environment triggered by senescent cells prevents adjacent cells from maintaining tissue homeostasis [13, 14] and it is proposed to induce senescence in a paracrine manner thus exacerbating tissue deterioration [15]. Currently, conventional pharmacotherapy for IVD degeneration has both a high cost and many potential negative side effects, which has stimulated the interest in natural plant-based products with anti-inflammatory and regenerative properties, as an alternative or adjunct to conventional therapy. These products are being investigated for potential efficacy in a wide range of disorders with an inflammatory component, including osteoarthritis and cancer [16, 17]. Recently, there have been a number of synthetic and natural drugs described with a specific mode of action to target and remove senescent cells, referred to as senolytics [18, 19].

Senolytics target many different pathways such as interfering with the dependence receptors, which promote apoptosis when unoccupied by ligands. Targeting and blocking signaling pathways involved in cell survival regulation interferes with mitochondrial-dependent apoptosis [20]. One natural senolytic, o-vanillin, a metabolite of a Curcumin, has anti-inflammatory properties and potent senolytic activity with a very wide non-toxic window for non-senescent IVD cells [18]. Treatment with o-vanillin has previously been shown to increase proteoglycan production of nucleus pulposus (NP) cells pellet culture [18]. Furthermore, o-vanillin interacts with a variety of cell surface receptors including toll-like receptors (TLR), Vanilloid, Chemokine, and Opioid receptors and could broadly reduce the levels of pro-inflammatory mediators and reduce matrix degradation, possibly preventing IVD degeneration [21, 22].

In human IVD’s there is expression of TLR-1, 2, 3, 4, 5, 6, 9, and 10 and the expression of TLR-1, 2, 4, and 6 are increased with degree of disc degeneration and pain [23]. Overloading of intact disc and IVD cells can upregulate TLR-2 and 4 expression, and previous data from our lab demonstrates that activating TLR receptors with the synthetic agonists (PAM2csk4, TLR-2/6 agonist) induced IVD degeneration [23–25]. Furthermore, studies using multiple cell lines proposed that TLR activation is associated with the induction of senescent cells and SASP factor release [26–28].

The present study investigates a possible link between the increase of TLRs and senescent cells in degenerate IVDs from patients undergoing surgery for low back pain. We show that a TLR-2/6 agonist increased the number of senescent cells from non-degenerate IVDs and in cells from degenerate IVDs. As well, we describe that TLR-2 has the highest expression and co-localization with senescent cells from degenerate IVDs from patients undergoing surgery for low back pain. Furthermore, treatment with o-vanillin reduced the number of cells co-localized for TLR-2 and senescence markers. From this study, we propose that TLR-2 has a role in the increase of senescent cells found in degenerating IVDs and that o-vanillin’s senolytic and anti-inflammatory activity could be a disease-modifying pharmaceutical for low back pain.

## Methods

### Tissue collection and cell isolation

All procedures performed were approved by the ethical review board at McGill University (IRB#s A04-M53-08B and A10-M113-13B). Non-degenerate IVDs from humans with no history of back pain were obtained through a collaboration with Transplant Quebec. Degenerate IVDs were obtained from patients with chronic low back pain that received discectomies to alleviate pain. Donor information is presented in Supplementary Table 1. IVD cells were isolated, as previously described [29]. Briefly, samples were washed in phosphate-buffered saline solution (PBS, Sigma-Aldrich, Oakville, ON, Canada) and Hank’s-buffered saline solution (HBSS, Sigma-Aldrich, Oakville, ON, Canada) supplemented with Primocin™ (InvivoGen, San Diego, CA, USA) and Fungiozone (Sigma-Aldrich, Oakville, ON, Canada). Then, the matrix was minced and digested in 0.15% collagenase type II (Gibco) for 16 h at 37 °C. Cells were passed through both a 100-μm filter and 70-μm filter, before being re-suspended in Dulbecco’s modified Eagle media (DMEM, Sigma-Aldrich, Oakville, ON, Canada) supplemented with 10% fetal bovine serum (FBS, Gibco), Primocin™, Glutamax (Oakville, ON, Canada), and maintained in a 5% CO_2_ incubator at 37 °C.

### In vitro cell culture and treatment

#### Monolayer culture

Experiments were performed with NP cells from non-degenerate IVDs and degenerate IVDs (NP and annulus fibrosus (AF) cells) within passage 1 to 2. Twenty thousand cells were seeded in 8-well chamber slides (Nunc™ Lab-Tek™ II Chamber Slide™ System) for immunocytochemistry experiments following treatment. Three hundred thousand cells were seeded in 6-well plates (Sarstedt, TC plate 6-well, Cell+, F) for ELISA and RNA extraction following treatment. All cells were left to adhere for 12 to 24 h and then serum-starved in DMEM with 1X insulin-transferrin selenium (ITS, Thermo Fisher, Waltham, MA, USA) for 6 h prior to treatment. To examine the effects of different treatments, healthy cells were treated with either 100 ng/ml Pam2CSK4 (TLR-2/6 agonist, Invivogen), 100 ng/mL Pam3CSK4 (TLR-1/2 agonist, Invivogen), or 5 μg/mL lipopolysaccharide (LPS) (TLR-4 agonist, Invivogen) for 6, 12, 24, and 48 h. Cells were either left untreated (negative control) or treated with 100 ng/mL of Pam2CSK4 for 48 h of which treatment with 100 μM o-vanillin (Sigma-Aldrich, Oakville, ON, Canada) was initiated in the last 6 h of incubation [18, 23, 30].

#### Pellet culture

Three hundred thousand cells/tube were collected by centrifugation at 1500 rpm for 5 min. Pellets were incubated in 1 mL DMEM, 2.25 g/L glucose (Sigma-Aldrich, Oakville, ON, Canada), 5% FBS, 5 μM ascorbic acid, 1% GlutaMAX, 0.5% Gentamicin (Thermo Fisher, Waltham, MA, USA) at 37 °C and 5% CO_2_. Pellets were left in DMEM for 4 days to form and stabilize (in pre-treatment media) and then treated with 100 μM o-vanillin (Sigma-Aldrich, Oakville, ON, Canada) for 4 days; meanwhile, pellets in the control group stayed in DMEM with vehicle 0.01% DMSO (Sigma-Aldrich, Oakville, ON, Canada). Following the treatment period, pellets from both groups were cultured for 21 days and their culture media was collected every 4 days and pooled as post-treatment media.

### Immunofluorescence

Monolayer cultures (20,000 cells/well in 8-well chambered slide) were washed with PBS, fixed with 4% paraformaldehyde (Thermo Fisher, Waltham, MA, USA), and blocked in PBS with 1% BSA (Sigma-Aldrich, Oakville, ON, Canada), 1% goat serum, and 0.1% Triton X-100 (Sigma-Aldrich, Oakville, ON, Canada) for 1 h. Pam2CSK4 treated cells were stained with primary antibodies specific to NGF (Santa Cruz, Dallas, TX, USA), *p16*^*Ink4a*^ (Cintec-Roche, Laval, Qc, CAN), IL1β, TNF-α, IL8, and TLR-2 (Abcam, Cambridge, Ma, USA) overnight at 4 °C. Healthy cells were treated with *p16*^*INK4a*^ and TLR-2 only. After washing, cells were incubated with the appropriate Alexa Fluor® 488 or 594-conjugated secondary antibody (Thermo Fisher, Waltham, MA, USA) for 2 h at room temperature, and then counterstained with DAPI for nuclear staining. Photomicrographs were acquired with a fluorescent Olympus BX51 microscope equipped with an Olympus DP71 digital camera (Olympus, Tokyo, Japan). Ten images of each condition per donor were analyzed and positive cell percentage was quantified by Fiji ImageJ (version: 2.1.0/1.53 c). Briefly, the number of cells stained positive for one of the target proteins (NGF, IL-1β, TNF-α, and IL-8) were counted and compared to the total number of cells positive for DAPI staining. For the double staining (TLR-2 and *p16*^*INK4a*^), the percentage of positive cells represents the ratio of the number of cells positively stained for either one of the 2 markers (TLR-2 and *p16*^*INK4a*^) divided by the total number of cells positively stained for DAPI.

### Immunohistochemistry

#### Safranin-O staining

Pellet culture samples were heated on an iron heater at 50 °C for 30 min and rehydrated with PBS. Samples were stained with 0.1% Safranin-O (Sigma-Aldrich, Oakville, ON, Canada) for 5 min at room temperature and rinsed with water, 75% ethanol (15 s), and 95% ethanol (15 s). Coverslips were mounted with Permount™ Mounting Medium (Fisher Scientific). Samples were imaged with Olympus DP70 digital camera (Olympus) pre-fixed to a Leica microscope (Leica DMRB) under visible light.

#### *p16*^*INK4a*^ staining

*p16*^*INK4a*^ staining was performed for both monolayer cultures and pellet samples. Only the pellet samples were heated on an iron heater at 50 °C for 30 min and rehydrated by PBS-T (0.1% Triton X-100) for 10 min. Both healthy monolayer cultures and pellet samples were blocked with hydrogen peroxide for 10 min, washed three times, and saturated with 1% BSA, 1% goat serum, and 0.1% Triton X-100 for 10 min. All samples were incubated at 4 °C overnight for *p16*^*INK4a*^ antibody (CINTec Kit, Roche) and PBS-T for negative control. The HRP/DAB Detection IHC Kit (Abcam, ab64264) was used for detection. Counting staining was applied with Meyer’s hematoxylin (Sigma-Aldrich, Oakville, ON, Canada) for 2 min. Samples were rinsed with water (30 s), 75% ethanol (15 s), and 95% ethanol (15 s) afterwards and coverslips were mounted with Permount™ Mounting Medium (Fisher Scientific). Images were captured as described [18] for Safranin-O staining, and analyzed with Fiji Image J (version 2.1.0/1.53c).

### Real-time quantitative polymerase chain reaction (RT-qPCR)

RNA was extracted using the TRIzol chloroform extraction method previously described [31]. Five hundred nanograms of RNA was then reverse transcribed using a qScript cDNA Synthesis Kit (Quanta Biosciences, Beverly, MA, USA) with an Applied Biosystems Verti Thermocycler (Thermo Fisher, Waltham, MA, USA). RT-qPCR was performed using an Applied Biosystems StepOnePlus machine (Thermo Fisher, Waltham, MA, USA) with PerfecCTa SYBR Green Fast Mix (Quanta Biosciences, Beverly, MA, USA). Primer sequences for TLRs, senescent markers, pain and inflammatory markers (IL-6, IL-8, p16, p21, TNF-α, CXCL-10, CXCL-1, GM-CSF, TGF-β, CCL-2, CCL-5, CCL-7, CCL-8, NGF, BDNF, IL-8, TLR-1,2,4,6) and the housekeeping gene (GAPDH) can be found in Supplementary Table 2. All reactions were conducted in technical triplicate, and fold changes in gene expression were calculated by using the 2^−ΔΔCt^method, after normalizing to actin and non-treated samples [32].

### Protein analysis

To determine the concentration of NGF, IVD cells were cultured in monolayer (250,000 cells/sample) and then lysed using 300 μL of Cell Lysis buffer (RayBiotech, Norcoss, GA, USA). Cell lysates were incubated for 48 h at room temperature and protein concentrations were determined using ELISA kits, according to the manufacturer’s instructions (RayBiotech, Norcoss, GA, USA). Cell culture media from degenerate IVD cells cultured in monolayer and in pellets was used to assess the concentrations of IL-6, IL-8, IL-1β, and TNF-α. One hundred and fifty microliters of monolayer culture media and pellet pre-treated and pooled post-treated media was used. ELISAs were performed as per the manufacturer’s instructions (RayBiotech, Norcoss, GA, USA). Colorimetric absorbance was measured with a Tecan Infinite M200 PRO (Tecan, Männedorf, Switzerland) spectrophotometer and analyzed with i-control 1.9 Magellan software (Tecan, Männedorf, Switzerland). Protein levels of the treated conditions and controls were then compared.

### Dimethylmethylene blue assay

Dimethylmethylene Blue (DMMB) assays were conducted as previously described [18] to quantify sulfated glycosaminoglycans (sGAG) in the conditioned media of IVD pellets with or without o-vanillin treatment. Chondroitin sulfate was used to generate the standard curve. Pooled post-treatment media samples from treated and untreated pellets were used. All samples were ensured to fall into the linear portion of the standard curve. Each sample was placed in triplicate into clear 96-well plates (Costar, Corning, NY, USA). DMMB dye was then added to the wells. The absorbance was measured immediately at room temperature using Tecan Infinite T200 spectrophotometer (Männedorf, Switzerland).

### Statistical analysis

Data was analyzed using Graph Prism 8 (Graph Pad, La Jolla, CA, USA). Analysis was performed using two-tailed Student’s *t*-test or two-way ANOVA. Specific tests are indicated in the figure legends with the corrections. A *p*-value < 0.05 was considered statistically significant. Data are presented as mean ± SD.

## Results

### Cell activation with a TLR-2/6 agonist, caused an increase in the number of senescent cells and SASP factor release in cells from non-degenerate human IVDs

**Fig. 1:**
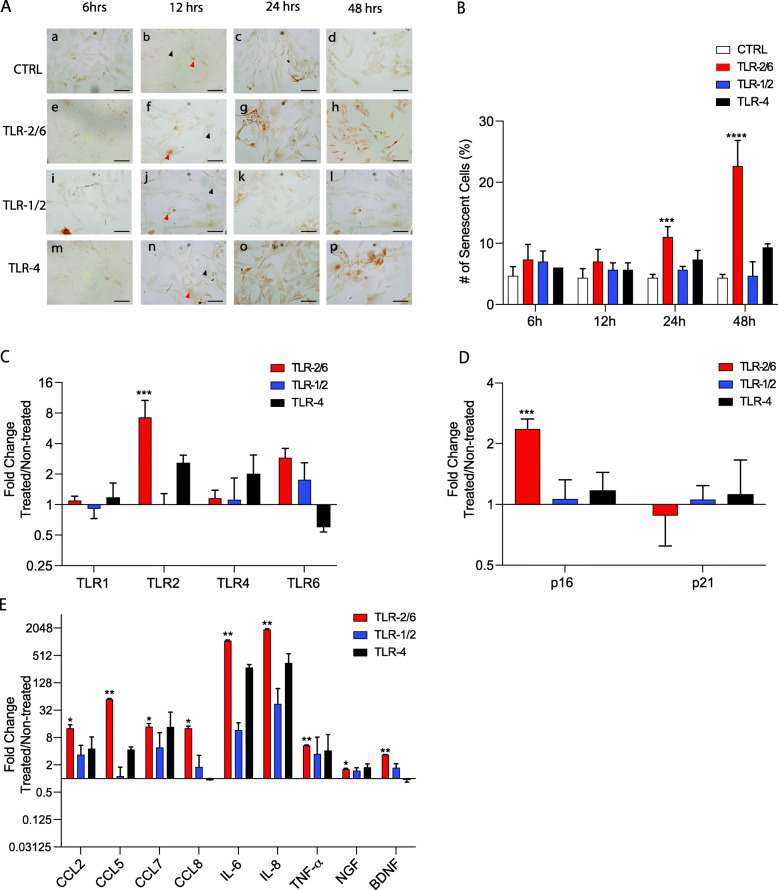
Cell activation with a TLR-2/6 agonist, caused an increase in the number of senescent cells and SASP factor release in cells from non-degenerate human IVDs. **A** *p16*^*ink4a*^ immunostaining images of untreated cells (**a**–**d**) from healthy donor IVDs or treated with either TLR-2/6 agonist (**e**–**h**), TLR-1/2 agonist (**i**–**l**), or TLR-4 agonist (**m**–**p**) cultured for 6, 12, 24, or 48 h. Scale bars = 25 μm. **B** Quantification of the percentage of *p16*^*ink4a*^ positive cells in the control and treated cells for the four time points. Examples of positive cell are indicated by the red arrow and for negative cells by the black arrow. **C**–**E** qPCR was performed using cells from healthy donor IVDs cultured for 48 h with TLR-2/6 agonist, TLR-1/2 agonist, or TLR-4 agonist. Gene expression for **C** TLR-1, -2, -4, and -6; **D** senescence markers *p16*^*ink4a*^ and p21; and **E** SASP factors (CCL2, CCL5, CCL7, CCL8, IL-6, IL-8, TNF-α, NGF. and BDNF). Fold changes were normalized relative to the non-treated control (CTRL). **B**–**E** Two-way ANOVA with Tukey’s multiple comparisons test, *n* = 5; significance was evaluated between treated groups compared to control group. Data shown as mean ± SD, *p < 0.05, **p < 0.01, ***p < 0.001, ****p < 0.0001

We have previously reported that TLR expression and the number of senescent cells are positively correlated with level of IVD degeneration [18, 23]. Moreover, we have seen that treating cells from degenerating human IVDs with TLR agonists PAM2csk4 (TLR-2/6), PAM3csk4 (TLR-1/2) and LPS (TLR-4) increased expression of pain mediators and pro-inflammatory cytokines when compared to vehicle control [23]. Here, we aimed to determine the effect and the relation of TLR activation and cell senescence in human IVD cells from non-degenerate IVDs. Monolayer cultures were treated with TLR agonists activating TLR-2/6, 1/2, and 4 for 6, 12, 24, and 48 h. p16^*ink4a*^ was used to identify senescent cells and the number of senescent cells were significantly increased following TLR-2/6 activation with a 11% ± 1.732 increase at 24 h (*p* < 0.001) and a 22.67% ± 4.163 (*p* < 0.0001) increase at 48 h (Fig. 1A, B). Furthermore, using RT-qPCR the gene expression levels of TLRs, senescence markers, and SASP factors were evaluated in the treated cell pellets. The expression of the TLR-1,2,4,6 in pellets treated with TLR agonists for 48 h was investigated. A significant increase (7.24-fold ± 3.458, *p* < 0.001) in expression of the TLR-2 in the cells exposed to the TLR-2/6 agonist was observed when normalized to the untreated control. Significance was not reached for TLR-1 (*p* = 0.9995), TLR-4 (*p* = 0.9974), and TLR-6 (*p* = 0.1080) (Fig. 1C). Moreover, a significant increase (2.56-fold ± 0.288, *p* < 0.001) of p16^*ink4a*^ gene expression only in cells exposed to the TLR-2/6 agonist when compared to control was observed. Of note, no significant difference was found in p21 gene expression following exposure to TLR2/6 agonist (*p* = 0.8557) (Fig. 1D). When evaluating SASP factors after 48 h of exposure to the TLR agonists, the most significant increase was observed following TLR2/6 exposure (Fig. 1E). Comparing to the control, an increase in CCL2 (12.70-fold ± 2.541, *p* < 0.05), CCL5 (55.11-fold ± 2.696, *p* < 0.01), CCL7 (13.78-fold ± 2.440, *p* < 0.05), CCL8 (12.68-fold ± 1.534, *p* < 0.05), IL-6 (1079.12-fold ± 43.135, *p* < 0.01), IL-8 (1890.28-fold ± 85.617, *p* < 0.01), TNF-α (5.30-fold ± 0.214, *p* < 0.01), NGF (3.49-fold ± 0.250, *p* < 0.05), and BDNF (3.31-fold ± 0.051, *p* < 0.01) was seen following TLR2/6 activation (Fig. 1E). Altogether, these results validate that activation of TLR-2/6 increases both the number of senescent cells and SASP factors produced in cells from non-degenerate IVDs.

### o-Vanillin reduced the number of senescent cells and enhanced proteoglycan production in cell pellet cultures from degenerate IVDs

**Fig. 2:**
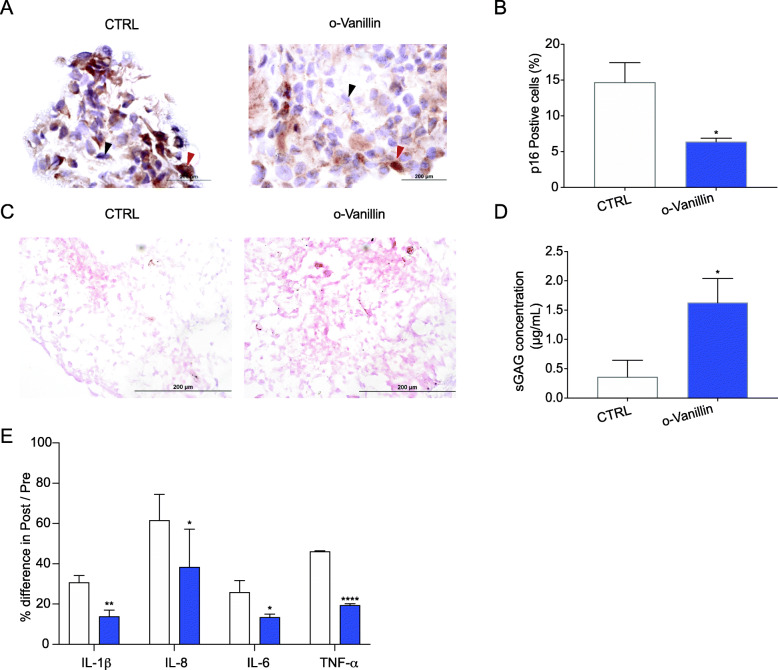
o-Vanillin reduced the number of senescent cells and enhanced proteoglycan production in cell pellet cultures from degenerate IVDs. All experiments were performed on day 21. **a** Representative photomicrographs of *p16*^*ink4a*^ immunohistochemistry staining in pellet cultures from degenerate IVD cells either treated with o-vanillin or not (CTRL). Examples of positive cell are indicated by the red arrow and for negative cells by the black arrow. **b** Quantification of the percentage of *p16*^*ink4a*^ positive cells in the pellet cultures; *n* = 3. **c** Images for Safranin O staining for proteoglycan content in CTRL and o-vanillin cell pellet cultures from degenerate IVD. **d** sGAG concentration measured using DMMB assay in the culture media of CTRL and o-vanillin cell pellets; *n* = 5. **e** Percentage of change in concentration of IL-1β, IL-8, IL-6, and TNF-α in pellet culture media from untreated and o-vanillin treated degenerate IVD cells measured by ELISAs. Percent difference was evaluated by normalizing the post-treated media to the pre-treated media; *n* = 5. *p < 0.05, **p < 0.01, ****p < 0.0001. **b**, **d**, **e** Mean ± SD, Statistical analysis was done using paired *t*-test

o-Vanillin senolytic activity and effect on matrix production has never been assessed on cells from patients with back pain and degenerating IVDs. Here we evaluated o-vanillin’s senolytic activity in 3D pellet cultures of IVD cells back pain patients. The pellet cultures were treated with o-vanillin (100 μM) or vehicle (DMSO 0.01%) for 4 days. At the end of the treatment period, the pellets were maintained in standard culture media for 21 days with the post-treatment media collection occurring every 4 days. The senolytic activity was evaluated by immunostaining for the senescence marker p16^*ink4a*^ (Fig. 2a). The percentage of p16^*ink4a*^ positive cells decreased significantly from 14.66% ± 2.758 in the untreated control to 6.38% ± 0.4973 in the o-vanillin treated pellets (*p* < 0.05) (Fig. 2b). Safranin-O staining was used to evaluate proteoglycan content. A more intense red staining was observed, indicating higher proteoglycan content in the o-vanillin treated IVD cell pellets compared to the control sample (Fig. 2c). Furthermore, a DMMB assay was performed to assess sGAG content in the culture media [33]. Pooled media from all post-treatment time points in the o-vanillin treated cell pellets (1.62 μg/ml ± 0.4134) had significantly higher sGAG content then the untreated control pellets (0.33 μg/ml ± 0.2876) (*p* < 0.05) (Fig. 2d). We then evaluated o-vanillin’s ability to reduce SASP factors (IL-1β, IL-8, IL-6, and TNF-α) that are commonly produced by senescent IVD cells [18]. Using ELISA immunoassay, we compared the percent difference of the pooled post-treated media over the pre-treated media. A significant decrease was observed in all evaluated SASP factors measured in the media of o-vanillin treated compared to the untreated controls. The percentage of difference in post compared to pre-treatment and measured in o-vanillin and control groups were respectively for IL-1β (13.75% ± 3.473 vs 30.63% ± 3.279, *p* < 0.01), IL-8 (38.38% ± 12.903 vs 61.5% ± 18.821, *p* < 0.05), IL-6 (13.38% ± 5.867 vs 25.75% ± 1.652, *p* < 0.05), and TNF-α (19.38% ± 0.408 vs 46% ± 0.750, *p* < 0.0001) (Fig. 2e).

### o-Vanillin reduced gene expression of p16, TLR-2, and SASP factors following TLR-2 activation in IVD cells from patients with back pain and IVD degeneration

**Fig. 3:**
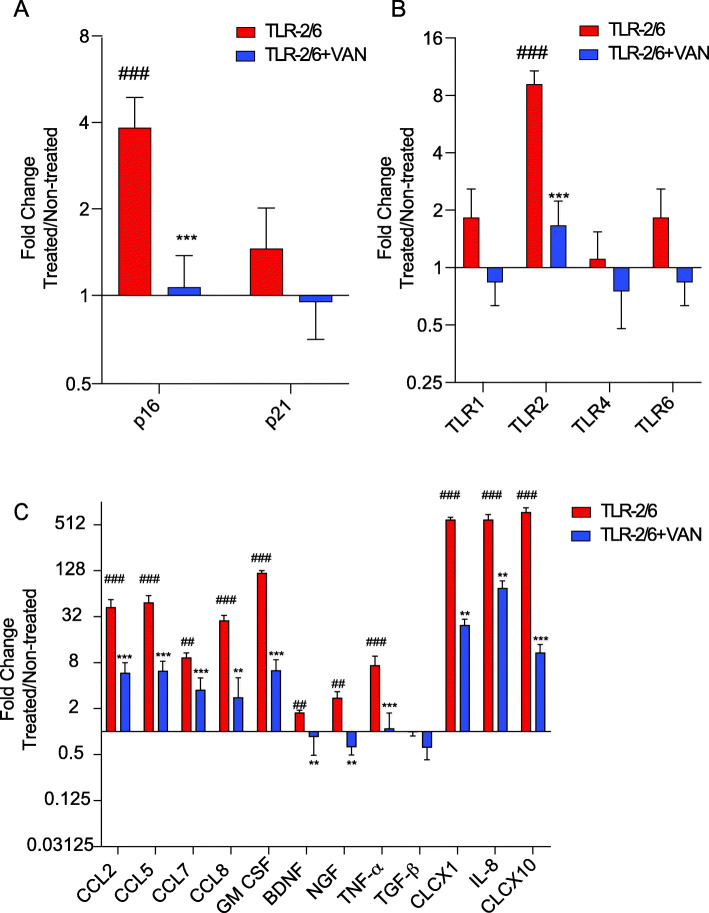
o-Vanillin reduced gene expression of p16, TLR-2 and SASP factors following TLR-2 activation in IVD cells from patients with back pain and IVD degeneration. **a**–**c** Gene expression of **a** senescence markers *p16*^*ink4a*^ and p21; **b** TLR-1, -2, -4, and -6; and **c** SASP factors (CCL2, CCL5, CCL7, CCL8, GM CSF, BDNF, NGF, TNF-α, TGF-β, CLCX1, CLCX8, CLCX10) of disc cells from degenerate IVDs cultured for 48 h with TLR-2/6 agonist with o-vanillin (TLR-2/6 + VAN) or without o-vanillin (TLR-2/6) treatment for 6 h or no induction with TLR-2/6. Fold changes were normalized relative to non-induced control. ^#^*p* < 0.05, ^##^*p* < 0.01, ^###^*p* < 0.001 indicate significant difference between the TLR-2/6 treated to the non-induced control and *p < 0.05, **p < 0.01, ***p < 0.001 indicate significant difference between groups TLR-2/6 + VAN and TLR-2/6. **a**–**c** Mean ± SD, measured by two-way ANOVA with Sidak’s multiple comparisons test. Values are expressed in average fold change for *n* = 5

IVD cells from patients with back pain and IVD degeneration were exposed the TLR-2/6 agonist for 48 h in the presence or absence of o-vanillin (100 μM) during the last 6 h of the treatment. We first assessed gene expression of senescence markers p16 and p21. Similar to the effect observed in cells from non-degenerate IVDs, there was a significant increase in p16^*ink4a*^ expression (3.83-fold ± 1.055, *p* < 0.001) and no significant difference in the expression of p21 (*p* = 0.4279) following TLR-2/6 exposure when compared to the untreated control (Fig. 3a). Interestingly, treatment with o-vanillin significantly decreased p16^*ink4a*^ expression (1.07-fold ± 0.308, *p* < 0.001), while no significant change was found for p21 expression (*p* = 0.244) (Fig. 3a). When assessing TLR expression in the cells from patients with back pain and IVD degeneration, a significant increase in TLR-2 gene expression (9.17-fold ± 1.594, *p* < 0.001) following TLR-2/6 exposure, compared to the control was seen. However, there was no significant increase in TLR-1 (*p* = 0.2420), TLR-4 (*p* = 0.9985), or TLR-6 (*p* = 0.3491) (Fig. 3b). These samples, when treated with o-vanillin showed a significant decrease (1.67-fold ± 0.565, *p* < 0.001) in TLR-2 expression (Fig. 3b). Exposure of IVD cells from patients with back pain and IVD degeneration to TLR-2/6 agonist significantly increased the expression of SASP factors CCL2 (42.32-fold ± 11.337, *p* < 0.001), CCL5 (49.03-fold ± 11.487, *p* < 0.001), CCL7 (9.30-fold ± 1.430, *p* < 0.01), CCL8 (28.40-fold ± 4.936, *p* < 0.001), GM-CSF (118.55-fold ± 10.067, *p* < 0.001), BDNF (1.77-fold ± 0.126, *p* < 0.01), NGF (2.75-fold ± 0.586, *p* < 0.01), TNF-α (7.36-fold ± 2.361, *p* < 0.001), CLCX1 (594.16-fold ± 44.718, *p* < 0.001), IL-8 (594.5-fold ± 98.644, *p* < 0.001), and CLCX10 (745.23-fold ± 107.787, *p* < 0.001) when compared to the untreated control (Fig. 3c). o-Vanillin significantly reduced this increase, CCL2 (5.89-fold ± 2.075, *p* < 0.001), CCL5 (6.21-fold ± 2.156, *p* < 0.001), CCL7 (3.51-fold ± 1.521, 0.001), CCL8 (2.8-fold ± 2.281, *p* < 0.01), GM-CSF (6.33-fold ± 2.39, *p* < 0.001), BDNF (0.85-fold ± 0.368, *p* < 0.01), NGF (0.62-fold ± 0.135, *p* < 0.01), TNF-α (1.09-fold ± 0.656, *p* < 0.001), CLCX1 (24.67-fold ± 5.132, *p* < 0.01), IL-8 (75.49-fold ± 18.608, *p* < 0.01), and CLCX10 (10.8-fold ± 3.087, *p* < 0.001) (Fig. 3c).

### o-Vanillin reduced the protein expression of SASP factors (IL-1β, NGF, IL-8, and TNF-α) following TLR-2/6 activation of IVD cells from patients with back pain and IVD degeneration

**Fig. 4:**
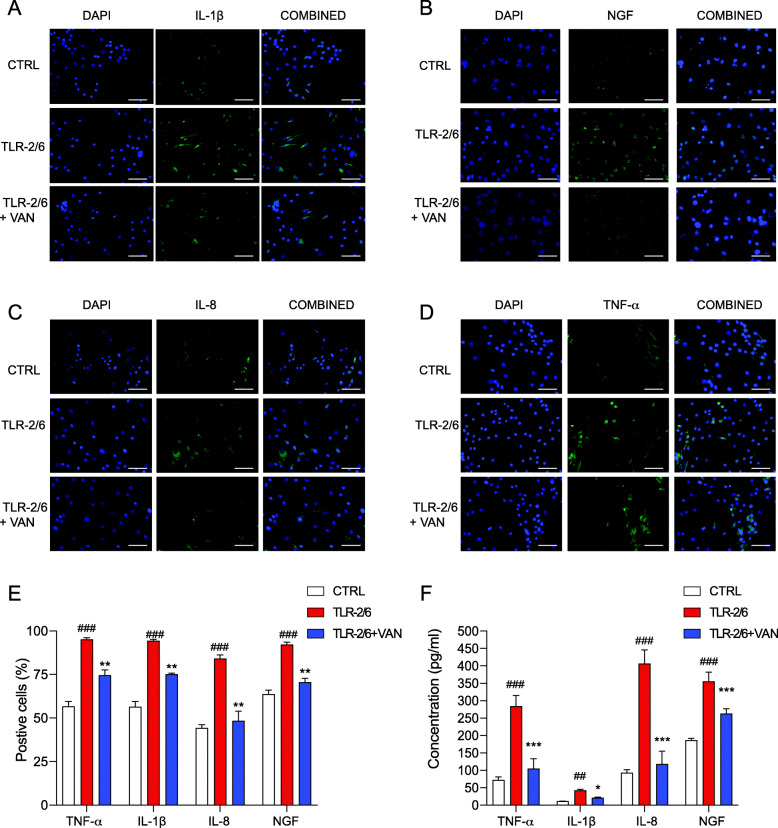
o-Vanillin reduced the protein expression of SASP factors (IL-1β, NGF, IL-8, and TNF-α) following TLR-2/6 activation of IVD cells from patients with back pain and IVD degeneration. Disc cells from degenerate IVD were cultured for 48 h with TLR-2/6 with o-vanillin (TLR-2/6 + VAN) or without o-vanillin (TLR-2/6) treatment for 6 h or no induction with TLR-2/6 (CTRL). **a**–**d** Using Immunocytochemistry, untreated and treated IVD cells were stained for DAPI and either **a** IL-1β, **b** NGF, **c** IL-8, and **d** TNF-α, respectively. Scale Bars = 25 μm. **e** Quantification of the percentage of the cells that stained positive for IL-1β, NGF, IL-8, and TNF-α when non-induced, treated with TLR-2/6 agonist or treated with TLR-2/6 agonist and o-vanillin; *n* = 5. **f** ELISAs were performed to measure the concentration of TNF-α, IL-1β, and IL-8 in cell media and NGF from cell lysate in non-induced, TLR-2/6 agonist treated or the combined treatment TLR-2/6 agonist and o-vanillin treated samples. Percentage of positive cells in **e** were the average for *n* = 5, . ^#^*p* < 0.05, ^##^*p* < 0.01, and ^###^*p* < 0.001 indicate significant difference between the TLR-2/6 agonist treated to the non-induced control and *p < 0.05, **p < 0.01, and ***p < 0.001 indicate significant difference between the tested groups. **e**, **f** Mean ± SD, statistical analysis was done using paired *t*-test

Protein expression of (TNF-α, IL-1β, IL-8, and NGF) was evaluated by immunohistochemistry following a 48-h exposure to TLR-2/6 agonist. (Fig. 4a–d). Quantification of the percentage of positive cells for each SASP factor was compared to untreated controls. A significant increase of TNF-α (56.6% ± 2.881 vs 95% ± 1.155, *p* < 0.001), IL-1β (56.4% ± 3.050 vs 94.25% ± 0.957, *p* < 0.001), IL-8 (44.2% ± 1.924 vs 84% ± 2.236, *p* < 0.001), and NGF (63.6% ± 2.408 vs 92.15% ± 1.388, *p* < 0.001) positive cells was observed (Fig. 4e). When evaluating the effect of o-vanillin, a significant decrease was observed in protein expression in the treated samples for TNF-α (74.5% ± 3.109, *p* < 0.01), IL-1β (75% ± 0.816, *p* < 0.01), IL-8 (48.4% ± 5.550, *p* < 0.01), and NGF (70.46% ± 2.416, *p* < 0.01) (Fig. 4e). Additionally, to measure the concentrations of SASP factors affected by TLR-2/6 activation and o-vanillin treatment, we performed ELISAs immunoassay of the culture media for TNF-α, IL-1β, IL-8, and on the cell lysate for NGF. A significant increase was found in the SASP factors in the culture media following TLR-2/6 activation, TNF-α (72.03 pg/ml ± 9.044 vs 284.25 pg/ml ± 30.972, *p* < 0.001), IL-1β (10.97 pg/ml ± 1.09 vs 42.11 pg/ml ± 3.022, *p* < 0.01), IL-8 (92.95 pg/ml ± 8.385 vs 406.25 pg/ml ± 39.891, *p* < 0.001) and in the cell lysate for NGF (186 pg/ml ± 5.957 vs 355.03 pg/ml ± 27.086, *p* < 0.001) when compared to the control and that this induction was significantly decreased for all evaluated SASP factors following treatment with o-vanillin (TNF-α, 104.5 pg/ml ± 28.831 (*p* < 0.001); IL-1β, 21.15 pg/ml ± 2.123 (*p* < 0.05); IL-8, 117.8 pg/ml ± 36.944 (*p* < 0.001); and NGF, 262.83 pg/ml ± 5.208 (*p* < 0.001)) (Fig. 4f).

### o-Vanillin reduced the number of cells co-expressing TLR-2 and p16^*ink4a*^ in cells exposed to TLR-2/6 agonist

**Fig. 5:**
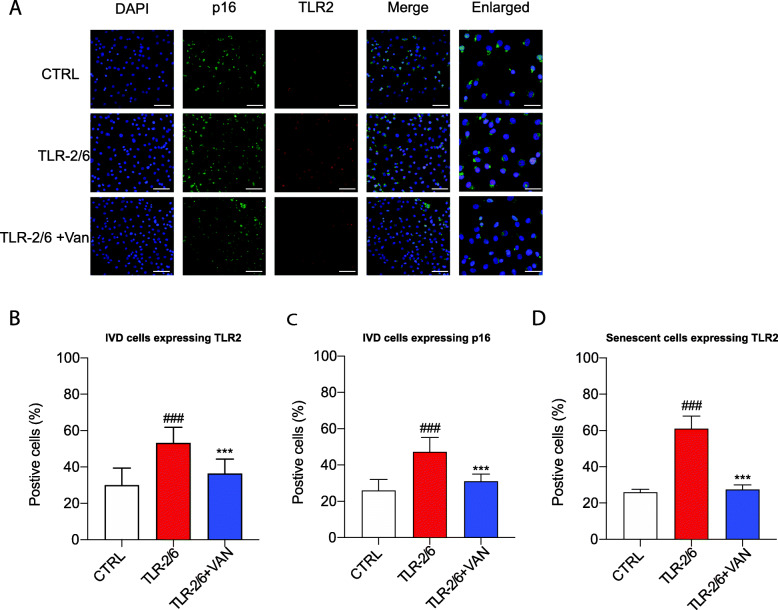
o-Vanillin reduced the number of cells co-expressing TLR-2 and p16^*ink4a*^ in cells exposed to TLR-2/6 agonist. Disc cells from degenerate IVDs were induced with TLR-2/6 agonist for 48 h with (TLR-2/6 + VAN) or without (TLR-2/6) o-vanillin treatment for 6 h or no induction with TLR-2/6 agonist (CTRL). **a** Photomicrographs of IVD cells stained for DAPI (blue) and either *p16*^*ink4a*^ (green), TLR-2 (red), or the merge (*p16*^*ink4a*^ and TLR-2) as revealed by Immunocytochemistry. DAPI, p16, TLR2, and merge images scale bars = 25 μm. Enlarged images scale bar: 10 μm. **b**–**d** Quantification of the percentage of IVD cells that stained positive for **b** TLR-2, **c** *p16*^*ink4a*^_,_ or **d** co-localized cell for TLR-2 and *p16*^*ink4a*^. Percentage of positive cells in **e** were the average for *n* = 5. ^#^*p* < 0.05, ^##^*p* < 0.01, and ^###^*p* < 0.001 indicate significant difference between the TLR-2/6 agonist treated to the non-induced control and *p < 0.05, **p < 0.01, and ***p < 0.001 indicate significant difference between the TLR-2/6 agonist with o-vanillin treated to the TLR-2/6 agonist treated. **b**–**d** Mean ± SD, statistical analysis was done using paired *t*-test

Based on our findings that exposure to TLR-2/6 agonist caused a significant increase in p16^*ink4a*^ and TLR-2 gene expression in both cell and pellet cultures from non-degenerate and degenerate IVDs, we investigated the possibility that senescent IVD cells have an elevated TLR-2 expression. Protein expression of TLR-2 and p16^*ink4a*^ was assessed by immunohistochemistry in IVD cells from patients with back pain and IVD degeneration following a 48-h exposure to TLR-2/6 agonist (Fig. 5a). Quantification of TLR-2 and p16^*ink4a*^ was done by measuring the percent of cells positive from the total cell population for the two markers. Following TLR-2/6 activation, it was found that there was a significant increase in the expression of TLR-2 (53.17% ± 8.684, *p* < 0.001) and p16^*ink4a*^ (47.19% ± 7.951, *p* < 0.001) when compared to untreated controls, TLR-2 (29.92% ± 9.448) and p16^*ink4a*^ (25.95% ± 6.071) (Fig. 5b, c). Furthermore, treatment with o-vanillin for the final 6 h significantly reduced this increase for TLR-2 (36.3% ± 8.057, *p* < 0.001) and p16^*ink4a*^ (31.07% ± 3.854, *p* < 0.001) (Fig. 5b, c). Finally, to verify the link between TLR-2 and cell senescence in IVD cells, we assessed the percentage of cells co-expressing p16^*ink4a*^ and TLR-2 by determining the percentage of senescent cells (p16^*ink4a*^ positive cell) that express TLR-2. In the untreated control, 26% ± 1.611 of the senescent cells expressed TLR-2 while following TLR2/6 exposure the percentage of senescent cells expressing TLR-2 increased significantly to 61.05% ± 6.946 (*p* < 0.001) (Fig. 5d). The most noteworthy finding was that o-vanillin significantly reduced the number of senescent cells expressing TLR-2 to 27.57% ± 2.509 (*p* < 0.001) when exposed to TLR-2/6 agonist (Fig. 5d). These findings indicate a link between TLR-2 expression, cell senescence, and SASP factor production that contribute to IVD degeneration and pain. This deleterious role of TLR-2 is blocked by the dual senolytic and anti-inflammatory effects of o-vanillin.

## Discussion

Several studies including our own have demonstrated that senescent cells accumulate in degenerating IVDs and suggested that an elevated SASP factor release and increased expression of TLRs contribute to IVD degeneration [18, 23]. Here, we have shown a potential link between the accumulation of senescent cells and TLR activation. As well, we show that o-vanillin, a TLR antagonist and senolytic compound, has regenerative and anti-inflammatory effects on cells from degenerating IVDs [34].

In chondrocytes and IVD cells, TLRs are, in addition to molecules derived from pathogens, activated by exposure to intracellular proteins such as HSP60, HSP70, S100A8/9, and HMGB1 released in response to stress and extracellular matrix fragments such as fibronectin, aggrecan, biglycan, and other by-products of tissue degeneration [35]. As well, it has been reported that synthetic TLR-2 and 4 agonists can induce IVD degeneration, increase inflammatory environment, and increase in expression of TLRs [23, 30]. The present study demonstrates that TLR-2 activation, in addition to inducing an inflammatory environment, caused IVD cells from non-degenerate IVDs to become senescent. We used cells of IVDs from organ donors with no signs of degeneration or history of back pain. These IVDs have a low number of senescent cells and low levels of SASP factor release compared symptomatic degenerating IVDs [18]. Our results demonstrate that the synthetic TLR2 agonist (Pam2CSK4) caused the greatest increase in senescent cell number, TLR-2 expression, and SASP factor release in cells from non-degenerate IVDs after 48 h exposure. Our previous study using TLR-1, 2, and 4 agonists found the cytokines (IL-1β, 6, 8), chemokines, proteases (MMP3, MMP13), and TLR-2 expression were greatest following exposure to the same TLR-2/6 agonist in NP cells of non-degenerate IVDs [23]. Other studies have shown that continuous stimulation of TLR-4 promotes cellular senescence in mesenchymal stem cells [36]. Moreover, TLR-2 and 10 have been found to be key mediators of senescence in IMR90 cells, a human diploid fibroblast cell line [26].

We then verified that these findings were also seen in cells isolated from degenerating IVDs of patients undergoing surgery to reduce low back pain [18, 29]. TLR-2 activation of cells from symptomatic IVDs induced expression of SASP factors (CCL-2,5,7,8, IL-6,8, GM-CSF, TNF-α, NGF, BNDF, CLCX-1,10), a senescence marker (p16^*ink4a*^) and of the TLR-2 receptor itself. Moreover, we confirmed that protein expression of SASP factors (NGF, IL-1β, TNF-α, and IL-8) was higher in the TLR-2 activated cells. These proteins were chosen since they have been associated to be IVD degeneration and TLR-2 induction and have been reported to be highly expressed in degenerate human and mice IVDs [30, 37, 38]. Taken together our results validate that IVD cells from patients with back pain and IVD degeneration at both gene and protein level respond to TLR-2 activation.

The use of synthetic antagonists aimed towards TLR-2 and TLR-4 has been evaluated in a variety of inflammatory diseases [39]. Antagonists such as TAK-242, a TLR-4 antagonist, has been shown to diminish LPS-induced TLR-4 signaling and inflammation in peritoneal macrophages [39]. Furthermore, our own previous study demonstrated that TAK-242 reduced pain but did not provide tissue regeneration in a mouse model of back pain [18]. Similar to our study, anti-inflammatory properties of o-vanillin was reported previously in NP cells from patients undergoing surgery for disc herniation or spinal stenosis following induction by high mobility group box-1 [40]. Furthermore, the capability of o-vanillin to reduce SASP factors has been previously depicted in IVD cell pellet cultures [18, 41]. o-Vanillin has also been shown to reduce cytokines, chemokines and proteases in vitro by in human HEK-TLR2 and THP-1 cells and to reduce a tumor-promoting phenotype of microglia in vivo [34, 42]. It has also previously been shown that o-vanillin incorporated to Poly (Lactic-*co*-Glycolic Acid) scaffolds elicited more proteoglycan production and decreased inflammatory response of annulus fibrous cells compared to cells in un-supplemented scaffolds [43]. As well, o-vanillin has been shown to significantly decrease the production of pro-inflammatory cytokines and significantly attenuated UVB irradiation-induced cytotoxicity in human keratinocyte stem cells [44].

Senolytic drugs target selective signaling pathways involved in cell survival and apoptosis [25]. These drugs could potentially be used therapeutically to treat disc degeneration, recover loss of disc height in already degenerate discs, or prophylactically to prevent future degeneration either in individuals at risk or following fusion for adjacent disc disease [45, 46]. Our previous study demonstrated that o-vanillin reduced senescence cells and enhanced matrix production in cell pellet cultures generated from organ donor IVDs without known history of back pain [18]. Here, we show that o-vanillin was able to reduce inflammation, remove senescent cells and enhance proteoglycan production in cell pellets from surgically removed symptomatic IVDs of patients with low back pain.

We further demonstrated that by targeting TLRs and senescent cells with o-vanillin, we can decrease inflammatory processes found in IVD cells from patients with back pain and IVD degeneration. Interestingly, our study demonstrates that both gene and protein expression of SASP factors (CCL2,5,7,8, GM-CSF, BDNF, NGF, TNF-α, CLCX1, CLCX8 and CLCX10, IL-1β, IL-8) were significantly reduced following TLR activation and o-vanillin treatment.

The higher expression of TLR-2 in IVD cells from patients with back pain and IVD degeneration leads us to evaluate its expression level in senescent cells and investigate its role in disc cell senescence and associated SASP factors release. We found TLR-2 activation increased the expression of TLR-2 in the senescent cells. Also, treatment with o-vanillin significantly reduced the number of senescent cells expressing TLR-2. One limitation of our study is that the degenerate cell population is a mix of NP and AF cells from patients suffering from chronic lower back pain. This is because the difficulty to accurately distinguish and separate NP and AF tissue from surgically removed IVD tissue. This limitation does not allow us to know whether the TLR-2/p-16 co-localization is in both cell types or in AF or NP cells specifically. To our knowledge, this is the first study to show a potential link between TLR-2 and cellular senescence in IVD cells. Further studies using genetically modified TLR-2 knock-out human IVD cell lines are needed to better decipher which mechanistic pathways are shared between o-vanillin’s senolytic activity and TLR-2’s antagonistic effect.

## Conclusions

We showed that TLR-2/6 activation increased TLR-2 expression and senescent cells in IVD cells from both organ donors without degeneration and back pain and patients with disc degeneration and back pain. Further, o-vanillin reduced the number of senescent IVD cells and the release of SASP factors. This data suggests a possible regulatory effect between TLR-2 and IVD cell senescence IVD. This phenomenon could be explained either by the induction of non-senescent neighboring cells by senescent cells in a paracrine manner or alternatively that senescent cells retain SASP factor production through TLR-2 activation in an autocrine manner. The detrimental effect of senescent cells can be inhibited by blocking TLR-2 activity with o-vanillin. These findings prompt the need to further understand the role of TLR-2 in IVD cell senescence and the mechanism by which o-vanillin interferes in this pathway.

## Supplementary Information

**Additional file 1: Supplementary Table 1.** Characteristics of the donors utilized for the study. (ICC): Immunocytochemistry including p16^*INK4a*^, immunofluorescence for NGF, IL-1β, TNF-α, IL-8, TLR-2 and p16^*INK4a*^ in a monolayer culture. (IHC): Immunohistochemistry for p16^*INK4a*^ and Safranin-O in pellet culture sections. (RT-qPCR): Real-time Quantitative Polymerase Chain Reaction (ELISA): Enzyme-linked immunosorbent assays. (DMMB): Dimethyl methylene blue (DMMB) assays.

**Additional file 2: Supplementary Table 2.** qRT-PCR Primer Sequences [47–52].

## Data Availability

The datasets used and analyzed during the current study are available from the corresponding author on reasonable request.

## Abbreviations

IVD: Intervertebral disc
TLR: Toll-like receptors
SASP: Senescence-associated secretory phenotype
DMMB: Dimethyl-Methylene Blue Assay
ICC: Immunocytochemistry
ELISA: Enzyme-linked immunosorbent assay
NP: Nucleus pulposus
AF: Annulus fibrosus
sGAG: Sulfated glycosaminoglycans
