# Supplementary material for: Toll-like receptor 2 induced senescence in intervertebral disc cells of patients with back pain can be attenuated by o-vanillin
Source: Arthritis Res Ther. 2021 Apr 16;23:117. doi: 10.1186/s13075-021-02504-z (PMC8051055; doi:10.1186/s13075-021-02504-z)
Supplement: Supplementary file 1 — Additional file 1: Supplementary Table 1. Characteristics of the donors utilized for the study. (ICC): Immunocytochemistry including p16INK4a, immunofluorescence for NGF, IL-1β, TNF-α, IL-8, TLR-2 and p16INK4a in a monolayer culture. (IHC): Immunohistochemistry for p16INK4a and Safranin-O in pellet culture sections. (RT-qPCR): Real-time Quantitative Polymerase Chain Reaction (ELISA): Enzyme-linked immunosorbent assays. (DMMB): Dimethyl methylene blue (DMMB) assays. [file 13075_2021_2504_MOESM1_ESM.docx]

**Supplementary Table 1.** **Characteristics of the donors utilized for the study.** (ICC): Immunocytochemistry including p16*^INK4a^*, immunofluorescence for NGF, IL-1β, TNF-α, IL-8, TLR-2 and p16*^INK4a^* in a monolayer culture. (IHC): Immunohistochemistry for p16*^INK4a^* and Safranin-O in pellet culture sections. (RT-qPCR): Real-time Quantitative Polymerase Chain Reaction (ELISA): Enzyme-linked immunosorbent assays. (DMMB): Dimethyl methylene blue (DMMB) assays.

| Donor | Age | Sex | Cause of death/ Reason For Surgery | ICC | IHC | RT-qPCR | ELISA | DMMB |
| --- | --- | --- | --- | --- | --- | --- | --- | --- |
| **Non-Degenerate IVD Donors** | | | | | | | | |
| 1 | 17 | M | Brain death | - |  | - | - |  |
| 2 | 20 | F | Intoxication CVA | - |  | - | - |  |
| 3 | 21 | M | Accident | - |  | - | - |  |
| 4 | 23 | F | Accident | - |  | - | - |  |
| 5 | 18 | M | Trauma | - |  | - | - |  |
| **Surgical samples** | | | | | | | | |
| 6 | 32 | M | Lower back pain and IVD Degeneration | - |  | - | - |  |
| 7 | 65 | F | Lower back pain and IVD Degeneration | - | - | - | - | - |
| 8 | 68 | F | Lower back pain and IVD Degeneration | - |  | - | - |  |
| 9 | 47 | F | Lower back pain and IVD Degeneration | - |  | - | - |  |
| 10 | 59 | F | Lower back pain and IVD Degeneration |  | - |  | - | - |
| 11 | 38 | F | Lower back pain and IVD Degeneration | - | - | - | - | - |
| 12 | 45 | M | Lower back pain and IVD Degeneration |  | - |  | - | - |
| 13 | 35 | F | Lower back pain and IVD Degeneration |  | - |  | - | - |
