# Supplementary material for: Toll-like receptor 2 induced senescence in intervertebral disc cells of patients with back pain can be attenuated by o-vanillin
Source: Arthritis Res Ther. 2021 Apr 16;23:117. doi: 10.1186/s13075-021-02504-z (PMC8051055; doi:10.1186/s13075-021-02504-z)
Supplement: Supplementary file 2 — Additional file 2: Supplementary Table 2. qRT-PCR Primer Sequences [47–52]. [file 13075_2021_2504_MOESM2_ESM.docx]

**Supplementary Table 2.** **qRT-PCR Primer Sequences**

| **Target** | **Forward Primer Sequence** | **Reverse Primer Sequence** | **Ref** |
| --- | --- | --- | --- |
| BDNF | 5’-TAACGGCGGCAGACAAAAAGA-3’ | 5’-GAAGTATTGCTTCAGTTGGCCT-3’ | (30) |
| CCL-2 | 5’-GCATGAAAGTCTCTGCCG-3’ | 5’-GAGTGTTCAAGTCTTCGGA-3’ | (47) |
| CCL-5 | 5’-GAAGGTCTCCGCGGCAGCC-3’ | 5’-CTGGGCCCTTCAAGGAGCGG-3’ | (47) |
| CCL-7 | 5’-CACTTCTGTGTCTGCTGCTCAC-3’ | 5’-GTTTTCTTGTCCAGGTGCTTCATA-3’ | (48) |
| CCL-8 | 5’-GCCTGCTGCTCATGGCAGCC-3’ | 5’-GCACAGACCTCCTTGCCCCG-3’ | (47) |
| CXCL-10 | 5’-GTGGCATTCAAGGAGTACCTC-3’ | 5’-TGATGGCCTTCGATTCTGGATT-3’ | (47) |
| IL-8 | 5’-TCCTGATTTCTGCAGCTCTG-3’ | 5’-GTCTTTATGCACTGACATCTAAGTTC-3’ | (18) |
| GAPDH | 5’-TCCCTGAGCTGAACGGGAAG-3’ | 5’-GGAGGAGTGGGTGTCGCTGT-3’ | (18) |
| GM-CSF | 5’-TCTCAGAAATGTTTGACCTCCA -3’ | 5’-GCCCTTGAGCTTGGTGAG-3’ | (49) |
| CXCL-1 | 5’TGAAGGCAGGGGAATGTATGTG -3’ | 5’-AGCCCCTTTGTTCTAAGCCA-3’ | (50) |
| IL-6 | 5’-TGAACCTTCCAAAGATGGCTG-3’ | 5’-CAAACTCCAAAAGACCAGTGATG-3’ | (18) |
| NGF | 5’-AAGTGCCGGGACCCAAAT-3’ | 5’-TGAGTTCCAGTGCTTTGAGTCAA-3’ | (23) |
| *p16^ink4a^* | 5’-CTGCCCAACGCACCGAATA-3’ | 5’-GCTGCCCATCATCATGACCT-3’ | (18) |
| *p21* | 5’-GAGACTCTCAGGGTCGAAAAC -3’ | 5’-GGCGTTTGGAGTGGTAGAAA-3’ | (18) |
| TGF- β | 5’-TCCTGGCGATACCTCAGCAA-3’ | 5’-CTCAATTTCCCCTCCACGGC-3’ | (51) |
| TLR-1 | 5’-CAGTGTCTGGTACACGCATGGT-3’ | 5’-TTTCAAAAACCGTGTCTGTTAAGAGA-3’ | (23) |
| TLR-2 | 5’-GGCCAGCAAATTACCTGTGTG -3’ | 5’-AGGCGGACATCCTGAACCT-3’ | (23) |
| TLR-4 | 5’-CAGAGTTTCCTGCAATGGATCA -3’ | 5’-GCTTATCTGAAGGTGTTGCACAT-3’ | (23) |
| TLR-6 | 5’-GAAGAAGAACAACCCTTTAGGATAGC -3’ | 5’-AGGCAAACAAAATGGAAGCTT-3’ | (23) |
| TNF-α | 5’-ATGTTGTAGCAAACCCTCAAGC-3’ | 5’-TCTCTCAGCTCCACGCCATT-3’ | (52) |
